# Supplementary material for: Attosecond spectroscopy of molecular charge transfer uncovers a 1.5-fs delay in population transfer
Source: Nat Commun. 2025 Aug 5;16:7211. doi: 10.1038/s41467-025-62162-6 (PMC12325650; doi:10.1038/s41467-025-62162-6)
Supplement: Supplementary file 1 — Supplementary Information [file 41467_2025_62162_MOESM1_ESM.pdf]

Supplementary Information for Attosecond spectroscopy of molecular charge transfer uncovers a 1.5-fs Delay in Population Transfer

Danylo T. Matselyukh[1,†], Florian Rott[2,†], Thomas Schnappinger[2,3,*], Pengju Zhang[1,4], Zheng Li[5,6], Jeremy O. Richardson[1], Regina de Vivie-Riedle[2], and Hans Jakob Wörner[1,*]

[1]Department of Chemistry and Applied Biosciences, ETH Zürich, 8093 Zürich, Switzerland
[2]Department of Chemistry, LMU Munich, 81377 Munich, Germany
[3]Department of Physics, Stockholm University, AlbaNova University Center, SE-106 91 Stockholm, Sweden
[4]Beijing National Laboratory for Condensed Matter Physics, IOP CAS, 100190 Beijing, People's Republic of China
[5]School of Physics, Peking University, 100871 Beijing, People's Republic of China
[6]Collaborative Innovation Center of Extreme Optics, Shanxi University, Taiyuan, Shanxi 030006, China
[†]These authors contributed equally to this work

Contents

1 Theoretical Details **2**
1.1 Origin and treatment of the asymmetric population transfer . . . 2
1.2 Simulating the 3-state model . . . 2
1.3 The Schrieffer–Wolf transform . . . 2

2 Experimental Details **4**
2.1 Experimental methods . . . 4
2.2 Extended analysis of TOF-MS results . . . 5
2.3 ATAS data acquisition and processing . . . 7
2.4 Fitting procedure for experimental results . . . 7

3 Computational Details **8**
3.1 Geometry of the Optimized Ground State Minimum . . . 8
3.2 Validation of the Electronic Structure Methods for CF_3I and CF_3I^+ . . . 8
3.3 Methodology for obtaining the XAS . . . 13
3.4 PECs and XAS of cationic Trifluoroiodomethane . . . 13
3.5 Discussion of the intermediate state(s) $\tilde{\mathrm{I}}$ . . . 17
3.6 Spectroscopic Assignment of the ground state XAS . . . 17
3.7 C–I bond length dependence of XAS cross-section . . . 18

4 Discussion on the absence of the $\tilde{\mathrm{A}}$-state signal **19**

5 Symmetry analysis of the CT reaction **21**

Supplementary references **23**

1 Theoretical Details

1.1 Origin and treatment of the asymmetric population transfer

The most basic crossing of two states in time is well understood within the Landau–Zener–Stueckelberg–Majorana (LZSM) treatment already introduced in the main text. Thanks to exact solutions of the problem as well as numerical and approximate treatments[1], it is know that the typical time evolution of the diabatic populations follows two phases; an initial jump in population which starts slow, but rapidly accelerates and reaches the asymptotic population. After this, a period of relaxation can follow, during which oscillations in population can sometimes be seen. One qualitative way of interpreting this evolution is to consider that the rate of population transfer depends on the population in the initial state; as the population of the initial state is rapidly depleted, so does the rate of population transfer.

As a result of the gradual start and rapid cessation of the dynamics, the rate of diabatic population transfer $\frac{dP_i}{dt}$ exhibits a clear asymmetry. Therefore, to achieve a robust fit of our simulated (and experimental) population dynamics the function used to fit them must possess an adjustable asymmetry. One of the most general functions of this type whose parameters are entirely uncoupled is the asymmetric generalized normal distribution (AGND)[2]. Its cumulative distribution function (CDF) takes the form

$$A(t) = \frac{a}{2}\left[1+\mathrm{erf}\left(\frac{y(t)}{\sqrt{2}}\right)\right]+c, \tag{S1}$$

where

$$y(t) = \begin{cases} -\frac{1}{\kappa}\log\left(1-\frac{\kappa(t-\mu)}{\sigma}\right) & \text{if } \kappa \neq 0 \\ \frac{t-\mu}{\sigma} & \text{if } \kappa = 0 \end{cases},$$

a is the amplitude, c is the offset, erf() is the error function, μ is the location parameter, σ is the scale parameter and κ is the shape parameter.

The asymmetry that the shape parameter κ describes is generally known as 'skew'. Functions with a long tail in the negative direction (as is the case for the rate of diabatic population transfer) are said to be 'negatively skewed' and exhibit $\kappa > 0$.

1.2 Simulating the 3-state model

For simulations occurring deep in the adiabatic regime, i.e. in the right two quadrants of Fig. 1D, the results were practically unchanged when propagating the wavepacket on the ground-state Born–Oppenheimer potential. We also found almost perfect agreement when using an ensemble of classical trajectories initialized from a Wigner transform of ψ_0 and evolved on the ground-state Born–Oppenheimer potential (see Fig. S1). At each timestep of the classical simulations, the diabatic populations were calculated using the square of the elements of the ground-state eigenvector of the diabatic Hamiltonian. These results suggests a direction that could be taken in future work to simulate a multi-state crossing and the associated delay from first principles.

1.3 The Schrieffer–Wolf transform

The Schrieffer–Wolf transform is able to decouple one sub-system from interactions with an energetically-distant sub-system. When applied to our model Hamiltonian, it can be used to find the effective 'direct' coupling between the initial $|0\rangle$ and final state $|2\rangle$.

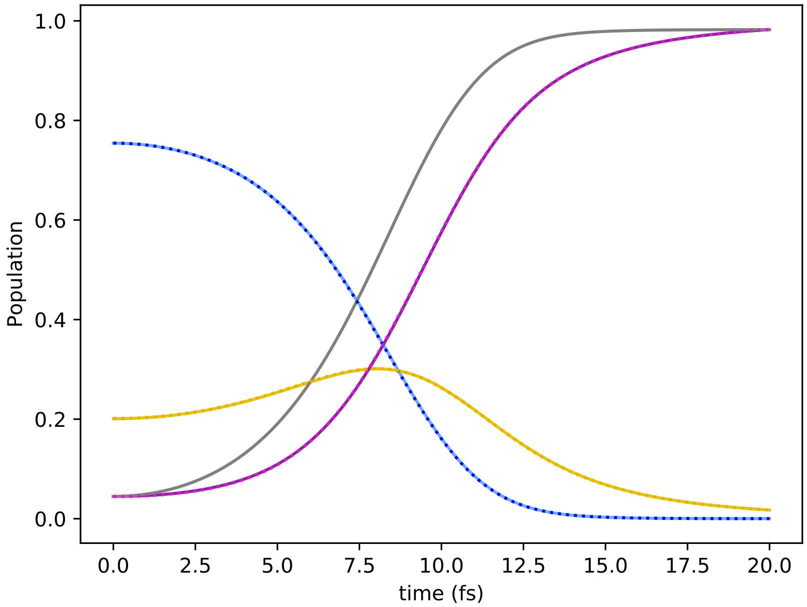

Figure S1. Comparison between the quantum and classical treatment of three-state model in the adiabatic limit. The parameters used for the simulations are the same as in Fig. 5. The line colors represent the three diabatic states; initial (blue), intermediate (yellow) and final (purple). The classical results are plotted as solid lines and the quantum results as dotted lines. The solid gray line shows the rescaled initial state population from the classical calculation. A population transfer delay is still observed.

To do this, the Hamiltonian $\hat{H}$ is first decomposed into a diagonal part $\hat{H}_0$ and the coupling

$$\hat{V}' = \begin{bmatrix} 0 & \gamma & 0 \\ \gamma & 0 & \gamma \\ 0 & \gamma & 0 \end{bmatrix}.$$

This coupling can be removed from the system Hamiltonian to first order in $\hat{V}'$ by finding a generator S that satisfies:

$$[\hat{H}_0, S] = \hat{V}', \tag{S2}$$

such that

$$\begin{aligned} \hat{H}' = e^S \hat{H} e^{-S} &= \hat{H} + [S, \hat{H}] + \ldots \\ &= \hat{H}_0 + \hat{V}' + [S, \hat{H}_0] + [S, \hat{V}'] + \ldots \\ &= \hat{H}_0 + [S, \hat{V}'] + \ldots \end{aligned}$$

Solving (S2) for S, we find

$$S = \begin{bmatrix} 0 & \frac{\gamma}{\alpha x - c} & 0 \\ \frac{-\gamma}{\alpha x - c} & 0 & \frac{\gamma}{\alpha x + c} \\ 0 & \frac{-\gamma}{\alpha x + c} & 0 \end{bmatrix}, \tag{S3}$$

allowing the Schrieffer–Wolf Hamiltonian to first order in $\hat{V}'$ to be found (see Equation 2 of the main text).

2 Experimental Details

2.1 Experimental methods

The optical setup of the experiment begins with a FEMTOPOWER V CEP laser system, which delivers 1.5 mJ, 25 fs laser pulses centered at 790 nm at a repetition rate of 1 kHz. These pulses are spectrally broadened in a 1 m long hollow-core fibre filled with neon, producing an octave spanning spectrum. Using eight bounces off PC-70 Ultrafast Innovations mirrors and a pair of fused silica wedges positioned at Brewster's angle to the beam, the supercontinuum is compressed to 5.2 fs. The duration of the pulse is characterized using a home-built second harmonic generation D-scan device.

The few-cycle pulse is used for both, generating the extreme-ultraviolet (XUV) isolated attosecond probe-pulse, and directly as a few-cycle pump-pulse. This is achieved by recycling the residual few-cycle driving field after the isolated attosecond pulse generation. The XUV attosecond pulse is generated through high-harmonic generation (HHG) in a differentially pumped finite gas cell filled with argon, after which the visible and XUV light is split using a drilled parabolic mirror. The reflected and collimated optical light then passes through a under-vaccuum, actively stabilized delay line, becoming the pump pulse. The 26 as stability of the delay line is achieved using two piezo-driving PID control loops for which the error signal is produced by a He:Ne based interferometer. The probe pulse is spectrally filtered using few-hundred-nanometer-thick aluminum foil which reflects the visible light while transmitting the XUV. A toroidal mirror is used to refocus the diverging XUV probe pulse into the transient absorption spectroscopy (TAS) target in a 2f-2f geometry, passing through another drilled parabolic mirror in the process. This parabolic mirror collinearly recombines the pump and probe-pulses, while also focusing the pump pulse into the TAS target.

Before running experiments on CF_3I, the XUV spectrometer was spectrally calibrated by measuring the Fano resonances in the photoionization continuum of neon which appear between 45 and 52 eV. Once

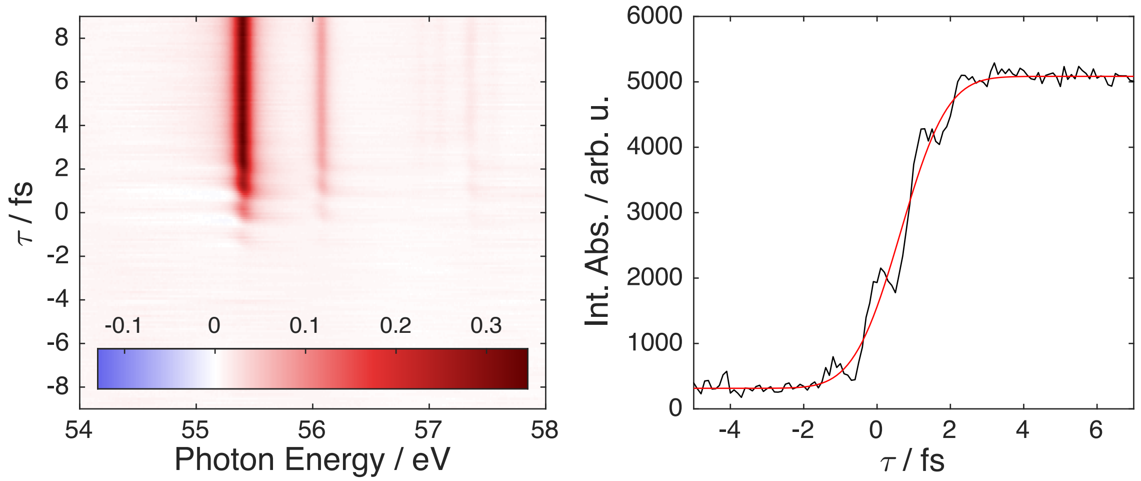

Figure S2. Determination of the instrument response function. Left: The results of ATAS on xenon atoms under experimental conditions very similar to the present work are shown in a pseudocolor plot; the measured change in optical density (ΔOD) is plotted as a function of photon energy and delay τ. The appearance of three absorption lines due to SFI of xenon are evident.Right: The integrated ΔOD over the strongest absorption line at 55.4 eV ($^2P_{3/2} \rightarrow ^2D_{5/2}$ of Xe^+, $5p^{-1}$) is fit with a error function determining the experimental cross-correlation standard deviation to be 1.00±0.05 fs. This figure is reproduced with permission from[3].

calibrated, the transient-absorption target was supplied with 99% purity CF_3I purchased from Apollo Scientific Limited and TAS was carried out by varying the delay between the pump and probe pulses using the delay line. The absolute delay was also simultaneously tracked with the help of a white-light interferometer. The pump intensity was varied with the use of a motorized iris placed in the delay line.

In addition to their XUV absorption spectrum, the ions generated by the pump pulse were also investigated with the help of a rudimentary home-built time-of-flight mass spectrometer (TOF-MS) installed into the transient-absorption chamber. By placing a positive potential on the TAS target body and positioning it 1 mm above the laser interaction region, the bottom of the TAS target doubles as a repeller that accelerates the strong-field ionized molecular fragments towards an ion detector. This detector takes the form of a Photonis MegaSpiraltron channeltron placed 16 cm below the interaction region. The field-free drift region of the TOF is achieved by placing a grounded mesh just above the channeltron and using a small diameter vacuum chamber, which through the Faraday-cage-effect, causes the potential of the TAS body to decay rapidly. A reflection mesh is used to redirect the ions into the channeltron once they have passed through the grounded mesh.

2.2 Extended analysis of TOF-MS results

The intensity-resolved results of the TOF-MS measurements are shown in Fig. S3. The peaks labeled in the TOF spectrum in Fig. 3D of the main text are integrated and plotted as a function of the laser peak intensity in Fig. S3A and B, with the latter being normalized to their highest abundance. From the un-normalized results, we can see that the three most abundant fragments generated by the strong-field ionization are CF_3I^+, CF_3^+ and, at intensities above 3×10^{14} Wcm^{-2}, I^+. These are the expected fragmentation products of the $\tilde{X}$, $\tilde{A}$ and $\tilde{B}$ states, respectively. Furthermore, the results show that the CF_3I^+ $\tilde{X}$ state and CF_3^+ $\tilde{A}$ state signals experience saturation effects and reach maxima at an intensity

around 4×10^{14} Wcm^{-2}. At these intensities, the few-cycle pump pulse not only depletes the CF_3I ground state population, but also starts to doubly ionize the sample, reducing the yield of the energetically lower-lying cationic states. These doubly ionized molecules are unstable and fragment by distributing the charge and breaking both the C-I and C-F bonds, producing the CF^+, CF_2^+ and even the F^+ fragments, or by concentrating the charge and producing the I^{2+} dication[4]. Intensities of 2 and 3×10^{14} Wcm^{-2} have therefore been used for our ATAS measurements to maximize the difference in the relative signal of the $\tilde{B}$ state dynamics while minimizing the contributions of higher-energy channels.

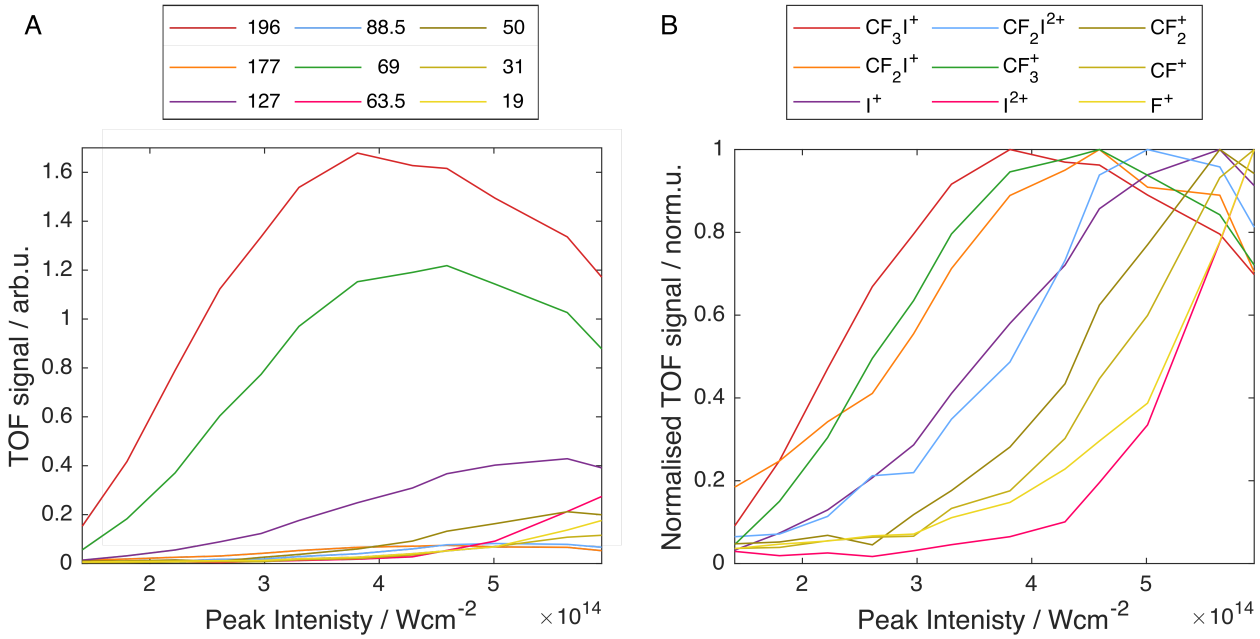

Figure S3. Intensity-resolved mass spectrometry on strong-field-ionized CF_3I. **A** The integrated ion yield of the different fragments as a function of ionizing-pulse intensity labeled according to their m/z ratio. **B** The integrated ion yields normalized to their maximum absolute yields, labeled with the chemical formula of the fragment.

The CF_2I^{2+} dication shows a similar intensity dependence as the I^+ cation, while exhibiting a 20-fold lower absolute fragmentation yield. This correlation with I^+ is peculiar as in previous strong-field ionization studies[4] using a 40 fs, 800 nm laser pulse, CF_2I^{2+} was determined to only originate from doubly ionized molecules. The absolute fragmentation was, however, far lower in the case of the narrower bandwidth 40 fs pulse, compared to our octave-spanning few-cycle pulse. The higher yield when using a broader spectrum indicates that the CF_2I^{2+} fragment may originate from an additional resonant electronic excitation of the $\tilde{B}$ state into the $\tilde{C}$ or $\tilde{D}$ states. These states are described by the ionization of an electron from non-fully-symmetric combinations of the fluorine F-orbitals, and are therefore capable of driving dynamics that break C-F bonds.

We note that this correlation-based analysis of the ion yields is performed on results obtained from a TOF-MS that was not purposefully built for the absolute determination of ion yields, but rather, the identification of ions. Nevertheless, we have demonstrated here that the insight gained from exploring the intensity-dependence TOF-MS is very powerful for determining coupled channels in strong-field ionization, even in the absence of a precise intensity calibration.

2.3 ATAS data acquisition and processing

A detailed description of the data-acquisition procedure is provided in section 2.1.9 of reference[3]. The ATAS results shown in Figs. 3-5 of the main text were acquired using the pulsed-nozzle-fed 3 mm tube target. Reference measurements at each delay were acquired by turning off the pulsed nozzle. A delay range of 100 fs was investigated using 500 as steps. The exposure time for each acquisition was set to 4 seconds and the measurement repeated three times (producing three 'scans').

To filter out the shot-to-shot fluctuations in the XUV spectrum of the probe pulse, a singular-value-decomposition- (SVD) based method (introduced in[5] and formally presented in section 2.3 of[3]) was used. Similar to other works in the field[6], it employs the correlations present in the data itself to filter out those signals that are uncorrelated with the delay.

The method decomposes the transient-absorption results of the three scans into the singular vectors. The reproducibility of the delay vector is then used to assess whether the vector describes true transient signals, or correlated fluctuations in the XUV spectrum. Those vectors that are not reproducible between scans fall into the latter category and are removed from the transient absorption results by having their singular values set to zero.

This filtration methodology requires the user to specify two parameters; the cut-off SVD index for filtration (beyond which the singular vectors are not filtered and not removed) and the cut-off reproducibility (the value of the reproducibility parameter below which a singular vector is removed). For the results presented in Figs. 4 and 5 of the main text, the cut-off index is set to 12 and the reproducibility cut-off to 0.1. The result of the SVD-based filtering is presented in Fig. S4.

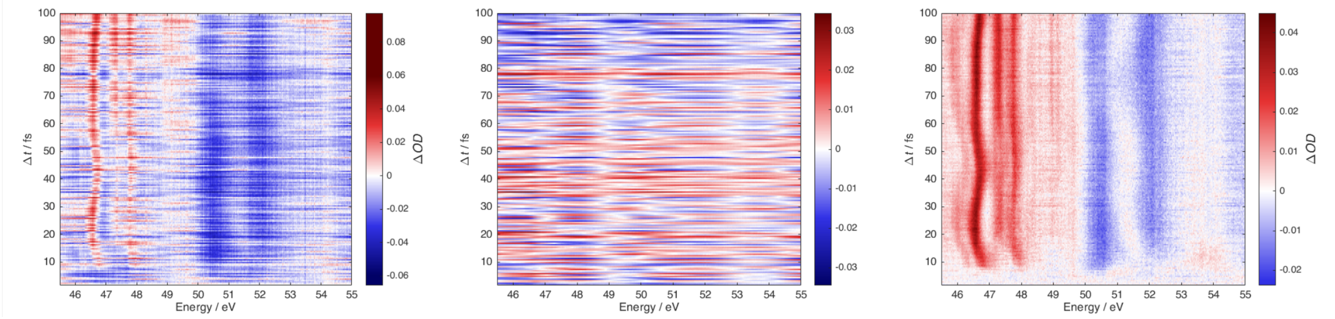

Figure S4. The result of the SVD-based filtration. The left panel shows the measured scan-averaged transient absorption. The middle column shows the signals originating from fluctuations in the XUV spectrum. The right-hand column shows difference between the first two panels: the SVD-filtered ATAS results.

2.4 Fitting procedure for experimental results

To maximize the robustness of the fits, we take every camera pixel which falls into the 47.25–47.36 eV and 53.50–54.30 eV photon-energy ranges to be an independent measurement of the diabatic population of the $\tilde{B}$ and $\tilde{E}$ states, respectively, and employ a bisquare-weighted regression. A smaller spectral range is chosen for the $\tilde{E}$ state than in Fig. 4A of the main text due to the fact that the $\tilde{X}_{3/2}$ state absorption extends slightly above 47.09 eV for $\Delta t < 8$ fs. The data and resulting fits are shown in Fig. 5A of the main text.

3 Computational Details

3.1 Geometry of the Optimized Ground State Minimum

The minimum geometry of the ground state of CF_3I was optimized with Gaussian 16[7] using density functional theory (DFT) with the ωB97X-D functional[8] and the basis set 6-311G[9,10]. The basis set was taken from the Basis Set Exchange (BSE)[11–13]. Table S1 shows the *xyz* coordinates of the optimized geometry in Ångström.

Table S1. Geometry of the ωB97X-D/6-311G optimized ground state minimum.

Number	Element	x (Å)	y (Å)	z (Å)
1	C	0.000000	0.000000	−0.004017
2	I	0.000000	0.000000	2.158913
3	F	1.242440	0.000000	−0.467045
4	F	−0.621220	1.075985	−0.467045
5	F	−0.621220	−1.075985	−0.467045

3.2 Validation of the Electronic Structure Methods for CF_3I and CF_3I^+

In order to sufficiently describe the valence excited states of CF_3I and CF_3I^+, three different active spaces (ASs) were tested. All calculations were carried out with the OpenMolcas[14,15] program package using the ANO-RCC[16–20] basis set, contracted to VDZP quality (ANO-RCC-VDZP). The smallest AS, forming the common basis for the two larger ASs, included 12 electrons in 10 orbitals [AS(12,10)]. It consisted of the carbon-iodine bond (σ_4, σ_5^*), both iodine lone-pair orbitals lp_1 and lp_2 as well as the three carbon-fluorine bonds (σ_1, σ_6^*, σ_2, σ_7^* and σ_3, σ_8^*). Subsequently, the medium AS was extended by three fluorine lone-pair orbitals lp_3, lp_4 and lp_5 resulting in 18 electrons in 13 orbitals [AS(18,13)]. The large AS was extended by an additional three orbitals (lp_6, lp_7 and lp_8), including all six fluorine lone-pair orbitals [AS(24,16)]. The orbitals included in the ASs are shown in Fig. S5. For the calculation of the cation, the same orbitals are included but with one electron removed, resulting in the set AS(11,10), AS(17,13) and AS(23,16).

In addition, density functional theory/multireference configuration interaction (DFT/MRCI)[21–23] calculations were performed to identify potential candidates for the proposed intermediate state $\tilde{I}$. In general, DFT/MRCI is a computationally efficient multireference approach to calculate a large number of excited states, including the necessary spin-orbit couplings fo the ionic states of interest. All DFT/MRCI calculations were performed with the GRACI program[24], using the QTP17[25] exchange correlation functional, the QE8 Hamiltonian[23] and the 6-311G** basis set. In total, 25 doublet and 10 quartet spin-free states were calculated giving rise to 90 spin-orbit coupled states, and a reference space of 24 Kohn-Sham orbitals (12 occupied and 12 virtual) were used for the MRCI step. The DFT/MRCI calculations were performed in a reproducible computing environment using the Nix package manager together with NixOS-QChem[26] (commit ee8700758).

As a first validation step for the three ASs and the DFT/MRCI results, the ionization energies were compared to the experimental values taken from the study of Yates and coworkers[27]. The states are order with respect to the results obtained using the largest active space AS(23,16). The ionization energies listed in Table S2 were calculated at the MS-CASPT2 and DFT/MRCI level of theory, including the effects of spin-orbit coupling (SOC), as the difference between the GS of the neutral CF_3I and the excited states

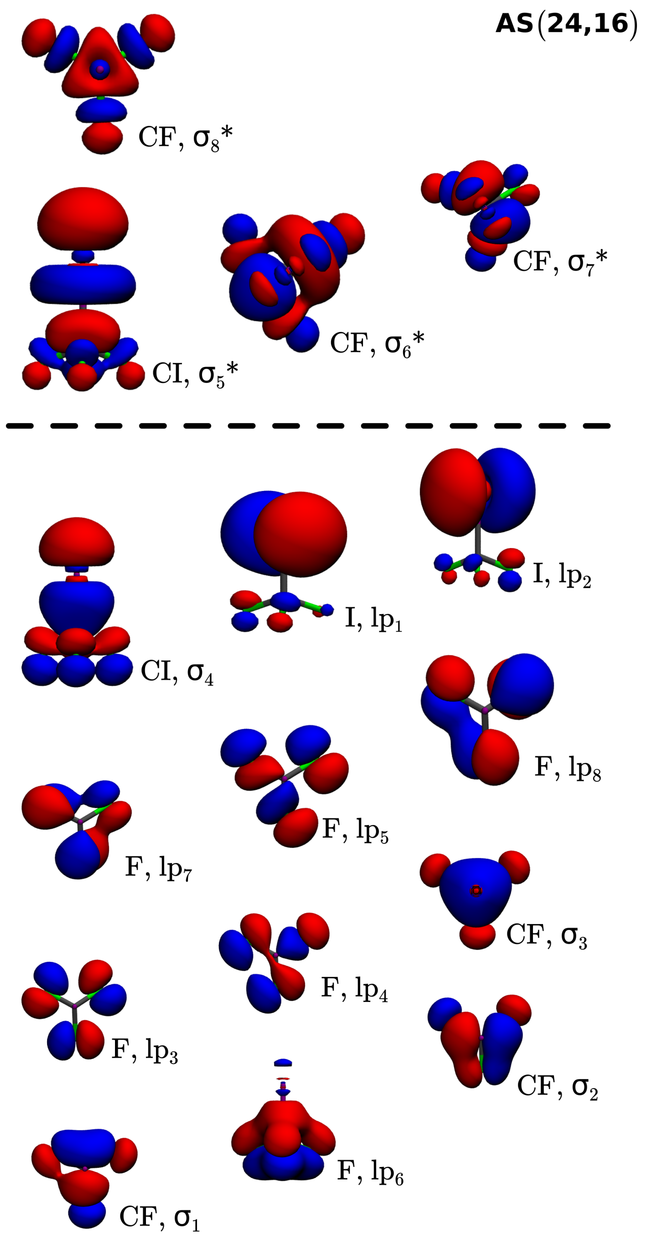

Figure S5. CASSCF molecular orbitals included in the active space AS(24,16) of trifluoroiodomethane, obtained using the ANO-RCC-VDZP basis set at the ωB79X-D/6-311G optimized ground state minimum geometry. Orbitals are rendered with an isovalue of 0.04. Orbitals σ_4, σ_5^*, lp_1, lp_2 and lp_6 are shown in a side view, whereas the rest are shown from the top along the C–I bond.

of the cation. Further, we compared the electronic character of the cationic states to calculated charge distributions taken from the same publication as listed in Table S3. The corresponding natural orbitals for the DFT/MRCI states are shown in Fig. S6. The first two states, $\tilde{X}$ $(^2E_{3/2})$ and $\tilde{X}$ $(^2E_{1/2})$, are a pair of spin-orbit coupled states described by an ionization from the I lone pair orbitals lp_1 and lp_2. Their ionization energies are described quite well by all three ASs and DFT/MRCI. The third state, $\tilde{B}$ $(^2A_1)$, an ionization from the C–I bonding σ_4 orbital, is correctly described by the AS(23,16) and DFT/MRCI, with the other two ASs overestimating the ionization energy. For the next three states, the electron hole is generated in the three F lone-pair orbitals lp_3, lp_4 and lp_5. These states cannot be described by the AS(11,10), as

the necessary orbitals are not included. The other two ASs correctly describe their electronic character and more or less capture the range of the ionization energy with increasing derivation for the higher lying states. The DFT/MRCI results for these three states ($\tilde{B}$, $\tilde{C}$, and $\tilde{D}$) consistently overestimate the ionization energy by approximately 0.3 eV and are thus in good agreement with the experimental value. For the largest AS(23,16) the state $\tilde{E}$ appears between the states $\tilde{B}$ and $\tilde{C}$. For the $\tilde{E}$ state the hole is again mainly on the I with the lp_1, lp_2 and σ_5^* orbitals partially occupied, which is probably why it could not be observed and described by Yates and coworkers[27]. Depending on the AS used and the electronic structure method, the energetic order of this $\tilde{E}$ state may vary. For all methods, it is above the $\tilde{B}$ state, but its position with respect to $\tilde{C}$ and $\tilde{D}$ can change.

Table S2. Ionization energies ΔE at the FC point for the first seven states of the CF_3I^+ cation in eV at the MS-CASPT2 level of theory, including spin-orbit couplings. The corresponding DFT/MRCI ionization energies are given in the last column. The energies were calculated as the difference of the GS energy of neutral species and the energies of the cationic states $\tilde{X}$-$\tilde{E}$. For the electronic character of the states, the partially occupied orbitals of the dominant configuration-interaction vector based on the calculation with the AS(23,16) are listed. The experimental ionization energies are taken from the work of Yates and coworkers[27]

State	Character	Ionization energies ΔE (eV)				
		AS(11,10)	AS(17,13)	AS(23,16)	Exp.[27]	DFT/MRCI
$\tilde{X}$ ($^2E_{3/2}$)	$lp_{1,2}$	10.74	10.49	10.54	10.45	10.40
$\tilde{X}$ ($^2E_{1/2}$)	$lp_{1,2}$	11.36	11.11	11.12	11.18	11.02
$\tilde{A}$ ($^2A_1$)	σ_4	14.07	13.74	13.30	13.25	13.00
$\tilde{B}$ ($^2A_2$)	lp_3	-	15.15	15.22	15.56	16.07
$\tilde{C}$ (^2E)	lp_4	-	17.01	15.92	16.32	16.73
$\tilde{D}$ (^2E)	lp_5	-	17.02	16.27	16.32	16.76
$\tilde{E}$ ($^2A_1$)	lp_1,lp_2, σ_5^*	16.33	16.18	16.80	-	16.14

Finally, the GS spectrum of neutral CF_3I was simulated for all three ASs following the procedure discussed in the manuscript and section 3.3 and compared with the experimental signal, as shown in Fig. S7. Here, all three spectra show the pronounced double-peak structures of the experiment at 50.4 eV and 52.1 eV and at 55.4 eV and 57.1 eV. But depending on the size of the AS, a different shift of the excitation energies needed to be applied. Shifting to the highest peak in the experiment at 50.4 eV, resulted in values of 2.00 eV, 1.60 eV and 1.30 eV for the spectra of RAS(22, 1, 1; 5, 7, 3), RAS(28, 1, 1; 5, 10, 3) and RAS(34, 1, 1; 5, 13, 3), receptively. In general, the prominent doublet at 50.4 eV and 52.1 eV is described quite well by all three ASs. With the RAS(34, 1, 1; 5, 13, 3) it was even possible to reproduce the correct intensity distribution of the said doublet. For the second doublet at 55.4 eV and 57.1 eV, all three spectra are energetically off by about 0.7 eV to 0.8 eV. Here, it is again possible that the size of the basis set ANO-RCC-VDZP prohibits a perfect match of this feature. However, considering the fact that calculations with a larger basis set were computationally infeasible and the otherwise excellent agreement with the experimental spectrum, we are convinced that the ASs (24,16) and (23,16) are adequate to describe the valence space and core-excited states of neutral and ionic CF_3I.

Table S3. Calculated charge distribution of the first seven cationic states of CF_3I^+ taken from the work of Yates and coworkers[27]. Out and In are the percentage charge of the outersphere and intersphere regions, respectively. The column Sum is not taken from the reference, but is an interpretation of the data. For our calculations the partially occupied orbitals of the transition with the largest configuration-interaction weight are shown as an approximation for the charge distribution.

	Charge distribution [%][27]						Partially occupied orbitals		
State	Out	C	F	I	In	Sum	AS(17,13)	AS(23,16)	DFT/MRCI
$\tilde{X}$ ($^2E_{3/2}$)	4.2	0.1	1.1	77.6	17.0	I^+	lp_1	lp_1	lp_1
$\tilde{X}$ ($^2E_{1/2}$)	4.2	0.1	1.1	77.6	17.0	I^+	lp_2	lp_2	lp_2
$\tilde{A}$ ($^2A_1$)	1.9	27.9	18.0	44.2	8.0	Non-local	σ_4	σ_4	σ_4
$\tilde{B}$ ($^2A_2$)	0.6	0.0	80.5	0.0	18.9	F^+	lp_3	lp_3	lp_3
$\tilde{C}$ (^2E)	0.7	0.3	78.4	0.3	20.3	F^+	lp_1,lp_2 σ_5^*	lp_4	lp_4
$\tilde{D}$ (^2E)	2.2	0.7	76.9	0.1	20.2	F^+	lp_4	lp_5	lp_5

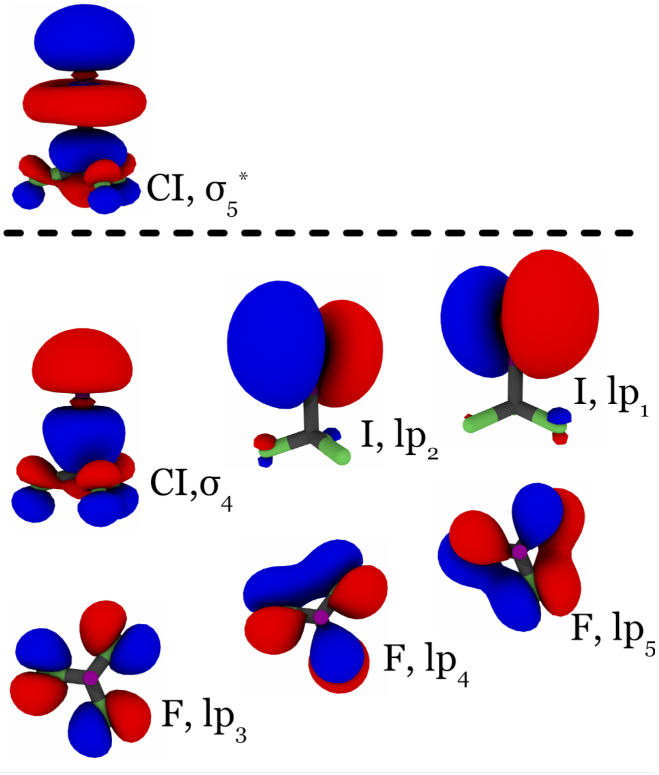

Figure S6. Natural orbitals of the DTF/MRCI calculations to describe all relevant ionic states. The orbitals were obtained using the 6-311G** basis set, the QTP17 exchange correlation functional in combination with the QE8 Hamiltonian. A reference space of 24 Kohn-Sham orbitals (12 occupied and 12 virtual) was employed for the MRCI step and the ωB79X-D/6-311G optimized ground state minimum geometry was used. All orbitals are rendered with an isovalue of 0.04. Orbitals σ_4, σ_5^*, lp_1 and lp_2 are shown in a side view, whereas the rest are shown from the top along the C–I bond.

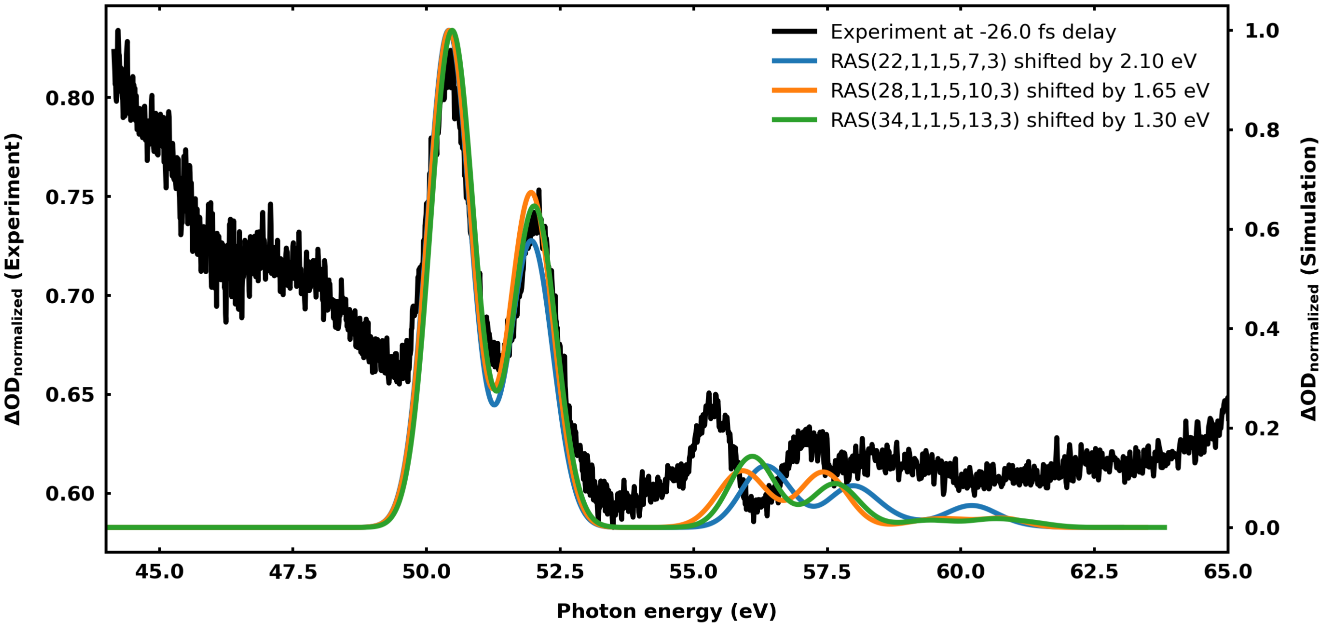

Figure S7. GS spectra of neutral CF_3I, simulated for the three different ASs. The calculated peaks are broadened by applying a Gaussian with $\sigma = 0.4\,\text{eV}$. In order to match the experimental signal shown in black, the excitation energies needed to be shifted by different amounts.

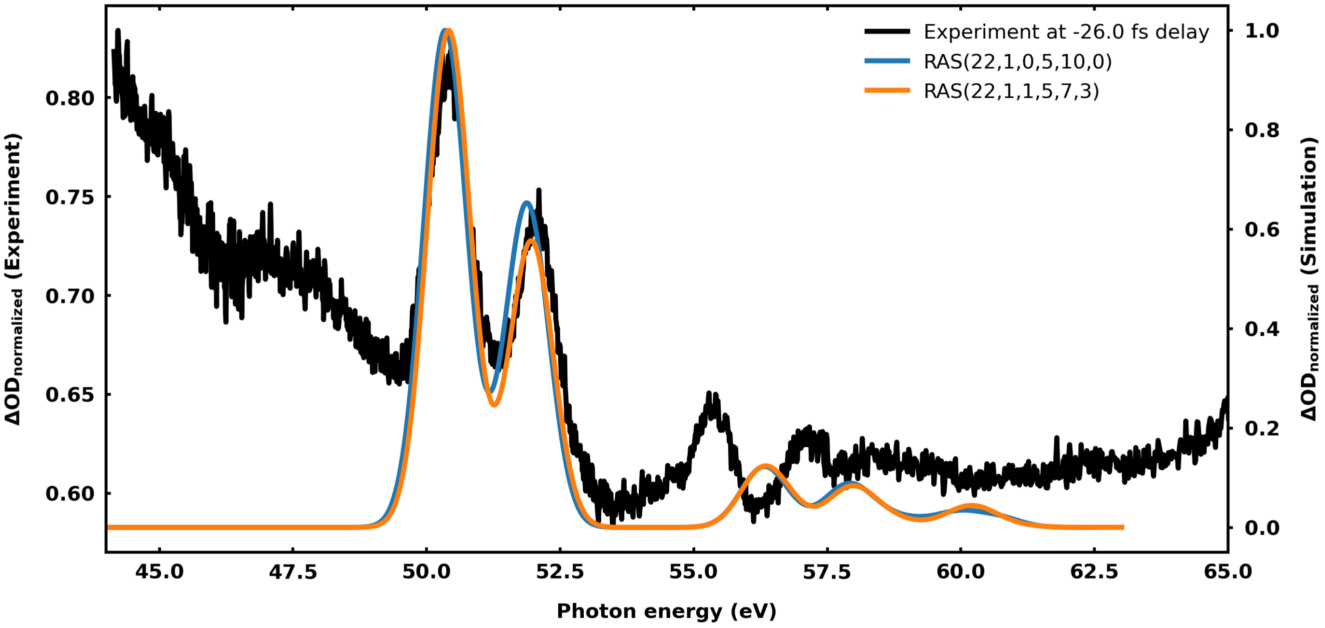

Figure S8. GS spectra of neutral CF_3I, simulated for the AS(12,10). In both cases, the excitation energies were shifted by $2.00\,\text{eV}$ to match the experimental signal shown in black and broadened by applying a Gaussian with $\sigma = 0.4\,\text{eV}$. The spectrum where all orbitals are included in the RAS2, is shown in blue. And the one where the AS is split up between the RAS3 and RAS2 sub spaces is shown in orange.

3.3 Methodology for obtaining the XAS

The general procedure on how to calculate an XUV absorption spectrum (also abbreviated as only XAS) based on the RASSCF/RASPT2 ansatz was introduced in detail in our previous study on the ultrafast strong-field dissociation of vinyl bromide[28]. So, here we only mention the aspects of the calculations that differ from the introduced procedure.

Based on the AS(24,16) shown in Fig. S5, the necessary RAS sub-spaces were set up for the calculation of the core-excited states. In general, the subspaces can be systematically labeled RAS$(n,l,m;i,j,k)$, where, i, j and k are the number of orbitals in the RAS1, RAS2, and RAS3 subspaces, respectively, n is the total number of electrons in the AS, l the maximum number of holes allowed in the RAS1, and m the maximum number of electrons allowed in RAS3. As the experiment probed at the $N_{4,5}$ edge of iodine, its five 4d orbitals were included in the RAS1. To reduce computational costs, the original AS(24,16) was split between the RAS2 and RAS3 subspaces. The three virtual orbitals of the carbon-fluorine bonds (σ_6^*, σ_7^* and σ_8^*) made up the RAS3 and a single excitation into them was allowed. The remaining 13 orbitals were included in the RAS2 subspace, resulting in the RAS$(34,1,1;5,13,3)$ illustrated in Fig. S9. To judge whether this influenced the simulated spectrum, we compared the GS spectrum of the 'full' AS with the approximation of splitting the AS. As this benchmark was performed for the AS(12,10), the resulting RAS are RAS$(22,1,0;5,10,0)$ and RAS$(22,1,1;5,7,3)$. Both calculated spectra, shifted by 2.00 eV to match the experimental signal, are shown in Fig. S8. The spectra are nearly identical, with the only difference in the intensity of the second peak of the doublet at 50.4 eV and 52.1 eV, which is slightly lower, when the AS is split between the two RAS subspaces. In summary, since the splitting of the AS does not significantly change the simulated spectrum and one can safely use this approximation, at least in the case of trifluoroiodomethane. But the total calculation time, necessary to arrive at the final spectrum, could be cut down drastically. The initial calculation time of over eight days for RAS$(22,1,0;5,10,0)$ could be reduced to about four and a half hours by utilizing the RAS3 sub-space. This speed-up made simulations with the AS(24,16) possible, as they could be completed in about three days. For the original setup of just using the RAS2 sub spaces, these calculations were not feasible as a total calculation time of over three months was estimated.

3.4 PECs and XAS of cationic Trifluoroiodomethane

To further analyze the dissociation dynamics of the cationic trifluoroiodomethane after excitation, we performed a relaxed scan of the C–I bond. The relaxed scan was performed with OpenMolcas utilizing the state tracking feature (keyword *TRACk*) of the *SLAPAF* program, where the character of a specific state can be followed throughout a geometry optimization. In our case, we chose to follow the character of the $\tilde{X}$ state at the Frank-Condon region. At each step in the scan, the geometry was optimized at the SA11-CASSCF(17,13)/ANO-RCC-VDZP level of theory. The C–I bond length was scanned from 1.6 Å to 5.0 Å with an initial step size of 0.05 Å but a bond length of 2.3 Å it was increased to 0.1 Å. In the Frank-Condon (FC) region between 2.0 Å to 2.3 Å the steps size was reduced to 0.025 Å. The resulting PECs are plotted in Fig. S10.

The electronic character of all relevant states, the partially occupied orbitals of the dominant configuration-interaction vector and its weight based on the calculation with the AS(23,16) along the relaxed scan are listed in Table S4.

Building on top of the optimized geometries we further performed single-point MS-RASPT2 calculations including spin-orbit effects utilizing the big AS(23,16) to obtain the XAS. To reduce the computational cost for these calculations, we put the three highest virtual orbitals (σ_6^*, σ_7^* and σ_8^*) into the RAS3 space and allowed for a single excitation [RAS(23,0,1,0,13,3)]. The results are shown in Fig. S14

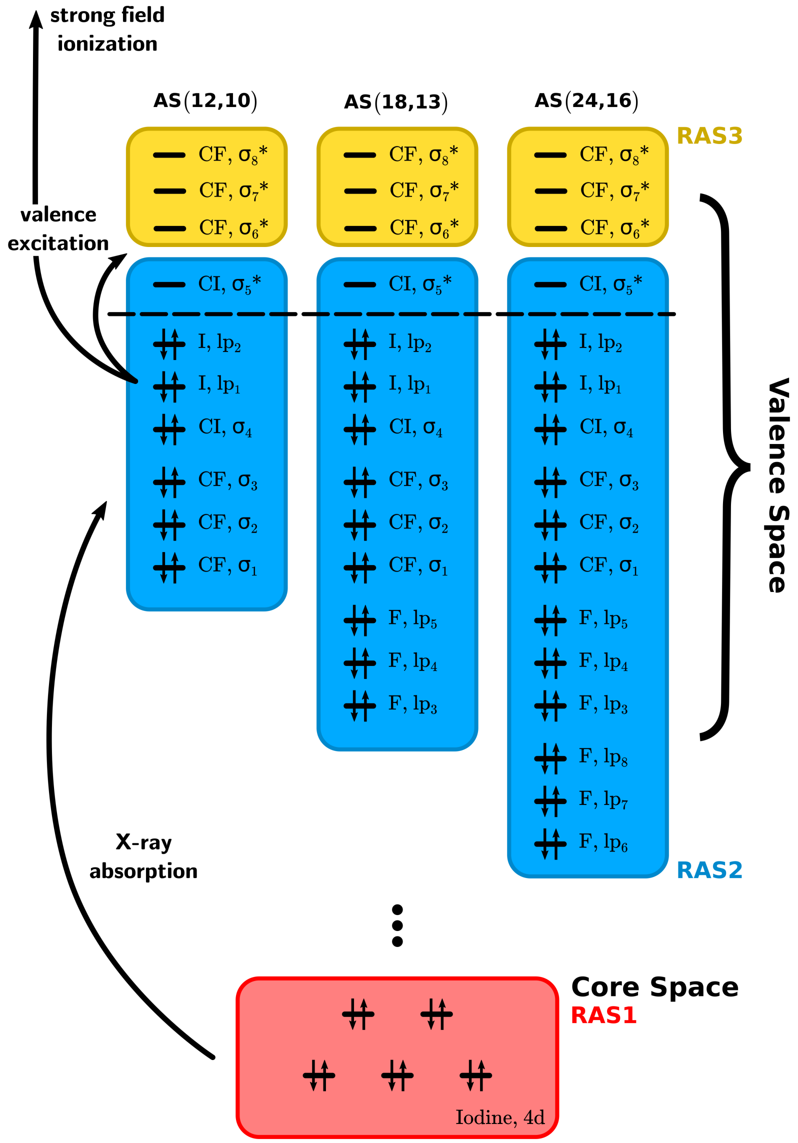

Figure S9. Diagram of the three active spaces RAS(22, 1, 1; 5, 7, 3), RAS(28, 1, 1; 5, 10, 3) and RAS(34, 1, 1; 5, 13, 3) tested for the simulation of the XAS of CF_3I. They are based on the previously introduced active space AS(12,10), AS(18,13) and AS(24,26).

as well as Fig. 3F and 4B of the main text. In these figures, an energy shift of 1.36, 1.36, 1.22, 0.98, and 0.98 eV have been applied to the $\tilde{X}_{3/2}$-, $\tilde{X}_{1/2}$-, Ã-, B̃- and Ẽ-states, respectively. The shift is determined by adjusting the position of the calculated XAS at the longest C-I bond length to best match the measured spectra of atomic iodine radicals and cations[29]. As the dissociation limit of the B̃-state is unknown, its shift is set to be the same as that of the Ẽ-state. The fact that this shift is unknown can lead to a larger mismatch between the calculated and experimental XAS of the B̃ state.

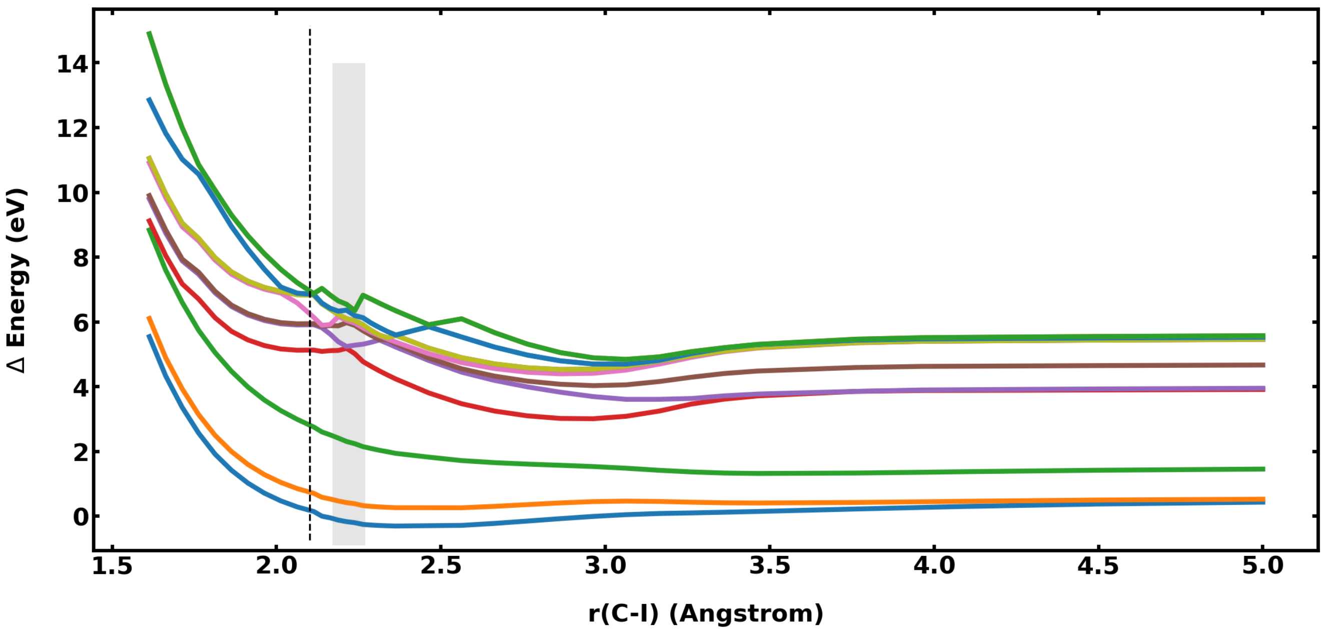

Figure S10. Relaxed scan of the C–I bond. The states are color-coded corresponding to their adiabatic state order. The FC region is indicated by the black dotted line and the gray box highlights the region of strong coupling.

Table S4. The electronic character of the seven relevant states at the optimized geometries of the relaxed scan of the C–I bond between 1.962 Å and 2.462 Å. For the electronic character of the states, the partially occupied orbitals of the dominant configuration-interaction vector and its weight in % based on the calculation with the AS(23,16) are listed.

	electronic character at R_{CI} (Å)									
#	1.962		2.012		2.062		2.112		2.137	
1	$lp_{1,2}$	52.8	$lp_{1,2}$	52.0	$lp_{1,2}$	92.0	$lp_{1,2}$	91.2	$lp_{1,2}$	94.5
2	$lp_{1,2}$	53.0	$lp_{1,2}$	52.3	$lp_{1,2}$	92.6	$lp_{1,2}$	92.2	$lp_{1,2}$	94.6
3	σ_4	92.3	σ_4	92.1	σ_4	92.0	σ_4	91.6	σ_4	92.3
4	lp_3	89.9	lp_3	89.3	lp_3	89.0	lp_3	88.4	lp_3	84.8
5	lp_4	65.3	lp_4	62.3	lp_4	60.0	lp_4	58.8	lp_4	83.5
6	lp_5	67.3	lp_5	64.9	lp_5	62.2	lp_5	60.0	lp_5	83.2
7	lp_7	67.2	lp_7	64.8	lp_7	61.9	lp_7	59.4	lp_1, lp_2, σ_5^*	87.0

	electronic character at R_{CI} (Å)									
#	2.162		2.187		2.212		2.237		2.262	
1	$lp_{1,2}$	94.6	$lp_{1,2}$	95.0	$lp_{1,2}$	95.0	$lp_{1,2}$	94.2	$lp_{1,2}$	94.5
2	$lp_{1,2}$	94.1	$lp_{1,2}$	95.1	$lp_{1,2}$	95.0	$lp_{1,2}$	94.3	$lp_{1,2}$	94.5
3	σ_4	92.1	σ_4	91.8	σ_4	91.9	σ_4	91.8	σ_4	89.1
4	lp_3	84.4	lp_3	80.4	lp_1, lp_2, σ_5^*	87.6	lp_1, lp_2, σ_5^*	87.2	lp_1, lp_2, σ_5^*	91.1
5	lp_1, lp_2, σ_5^*	86.4	lp_1, lp_2, σ_5^*	86.7	lp_3	80.8	lp_3	76.5	lp_1, σ_4, σ_5^*	57.6
6	lp_4	82.6	lp_4	74.4	σ_5^*	29.5	lp_2, σ_4, σ_5^*	51.0	lp_2, σ_4, σ_5^*	53.0
7	lp_5	82.6	lp_1, lp_2, σ_5^*	81.4	lp_1, lp_2, σ_5^*	67.8	lp_1, σ_4, σ_5^*	44.2	σ_5^*	38.0

	electronic character at R_{CI} (Å)									
#	2.287		2.312		2.337		2.362		2.462	
1	$lp_{1,2}$	94.2	$lp_{1,2}$	94.0	$lp_{1,2}$	93.8	$lp_{1,2}$	93.6	$lp_{1,2}$	92.9
2	$lp_{1,2}$	94.2	$lp_{1,2}$	94.0	$lp_{1,2}$	93.8	$lp_{1,2}$	93.7	$lp_{1,2}$	92.9
3	σ_4	89.0	σ_4	88.9	σ_4	88.8	σ_4	88.8	σ_4	88.6
4	lp_1, lp_2, σ_5^*	91.0	lp_1, lp_2, σ_5^*	90.7	lp_1, lp_2, σ_5^*	90.6	lp_1, lp_2, σ_5^*	90.4	lp_1, lp_2, σ_5^*	90.1
5	lp_1, σ_4, σ_5^*	67.0	lp_1, σ_4, σ_5^*	67.7	lp_1, σ_4, σ_5^*	68.1	lp_1, σ_4, σ_5^*	68.0	lp_1, σ_4, σ_5^*	66.5
6	lp_2, σ_4, σ_5^*	67.5	lp_2, σ_4, σ_5^*	68.0	lp_2, σ_4, σ_5^*	68.4	lp_2, σ_4, σ_5^*	68.2	lp_2, σ_4, σ_5^*	66.5
7	σ_5^*	43.3	lp_1, lp_2, σ_5^*	86.6	lp_1, lp_2, σ_5^*	90.6	lp_1, lp_2, σ_5^*	91.3	lp_1, lp_2, σ_5^*	73.2

3.5 Discussion of the intermediate state(s) Ĩ

As discussed in the main text, the CT reaction is likely to proceed through one (or more) diabatic intermediate state(s), collectively called Ĩ. By conceptually splitting the rearrangement into two single-electron transitions, we can identify the leading electronic characters of the possible intermediate states, which are $(\mathrm{lp}_3)^2(\sigma_4)^2(\mathrm{lp}_{1,2})^3(\sigma_5^*)^0$ and $(\mathrm{lp}_3)^1(\sigma_4)^2(\mathrm{lp}_{1,2})^3(\sigma_5^*)^1$. The first possible leading electronic configuration describes the electronic ground state of CF_3I^+, which is unlikely to act as intermediate state because we have not obtained experimental evidence of its transient population. The states characterized mainly by the second configuration should have significantly higher energy than the states accessible in our MS-CASPT2 calculations, however, it is possible to obtain these states using the DFT/MRCI method. By carefully analyzing all 90 spin-orbit coupled states calculated at the FC point using DFT/MRCI, we were able to identify a total of two possible candidates for Ĩ. Their ionization energy is shown in Table S5. The lower lying state $\tilde{I}_1$ is strongly coupled to a pair of quartet states of the same character and therefore

Table S5. Ionization energies ΔE at the FC point for four states of the CF_3I^+ cation relevant for the observed delay in the charge transfer mechanism given in eV at the DFT/MRCI level of theory, including spin-orbit couplings. The energies were calculated as the difference of the GS energy of neutral species and the energies of the cationic states $\tilde{X}$-$\tilde{E}$. For the electronic character of the states, the natural orbitals given in Fig. S6 are listed.

State	Character	Ionization energies ΔE (eV)
$\tilde{B}$ ($^2A_2$)	lp_3	16.07
$\tilde{E}$ ($^2A_1$)	lp_1, lp_2, σ_5^*	16.13
$\tilde{I}_1$ (^2E)	$\mathrm{lp}_{1,2}$, lp_3, σ_5^*	20.43
$\tilde{I}_2$ (^2E)	$\mathrm{lp}_{1,2}$, lp_3, σ_5^*	20.88

formally consists of four degenerate states. The other intermediate state $\tilde{I}_2$ is two times degenerate and has an almost pure doublet character. Both $\tilde{I}$ states are characterized by three single-occupied orbitals, as predicted by our preliminary considerations, and also fulfill the symmetry requirements that we discuss below in Section 5.

In addition to the single-point calculation at the FC point, we also constructed pseudo-diabatic PECs around the FC region for all relevant states, including the intermediate states shown in Fig. S11. The PCEs were calculated at the DFT/MRCI level of theory using the optimized geometries of the relaxed scan shown in Section 3.4. The DFT/MRCI PECs of the lower ionic states $\tilde{X}$ to $\tilde{E}$ are in good agreement with the CASSCF/CASPT2 results. We were able to identify the two intermediate states $\tilde{I}_1$ and $\tilde{I}_2$ for each C–I bond distance shown, and the energy spacing between these states and the states $\tilde{B}$ and $\tilde{E}$ are similar around the conical intersection (CoIn) region.

3.6 Spectroscopic Assignment of the ground state XAS

In the following, the features of the static GS spectrum of neutral CF_3I are assigned at the FC geometry. In the experimental signal shown in Fig. S7, two distinct doublets at 50.4 eV and 52.1 eV and 55.4 eV and 57.1 eV, in both cases with a splitting of 1.70 eV, are visible. When only a small shift of 1.30 eV to the excitation energies, the simulated spectrum matches the experimental one very well. The first doublet at 50.4 eV and 52.1 eV can be attributed to an excitation from the I 4d orbitals into the σ_5^* anti-bonding orbital of the C–I bond. The splitting of 1.70 eV arises from the SO splitting of the $4d_{3/2}$ and $4d_{5/2}$ orbitals

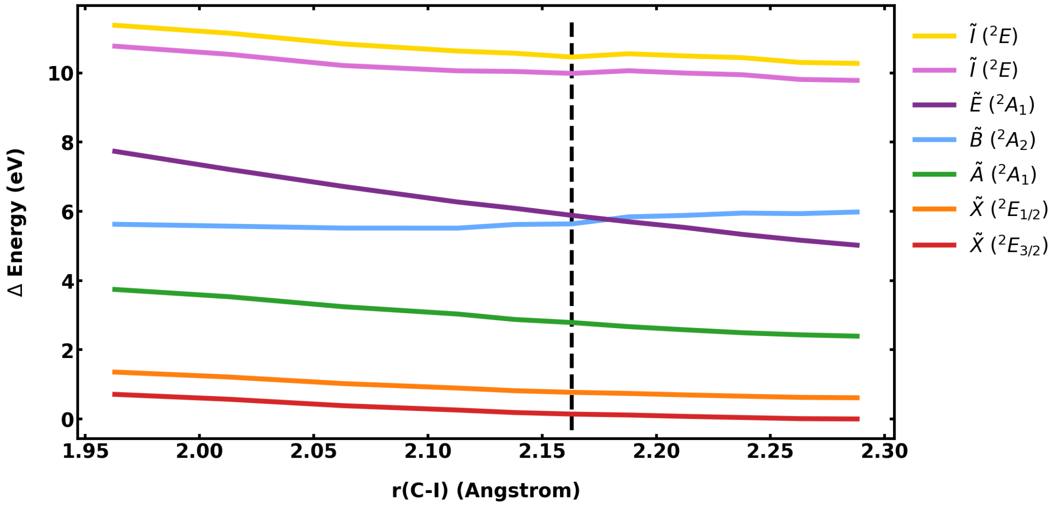

Figure S11. Pseudo-diabatic PECs around the FC region indicated by the black dotted line. The PCEs where calculated at the DFT/MRCI level of theory utilizing the optimized geometries of the relaxed scan shown in Section 3.4

of I. Similarly, the second doublet at 56.1 eV and 57.7 eV can be attributed to an excitation into the anti-bonding orbitals σ_6^*, σ_7^* and σ_8^* of the C–I bond. However, compared to the experiment, this doublet is shifted by about 0.7 eV to higher energies. This can be explained by the moderate size of the basis set ANO-RCC-VDZP, which may not be able to fully describe the σ_6^*, σ_7^* and σ_8^* orbitals, especially if these orbitals are subject to Rydberg-type contributions. Overall, the simulated steady-state spectrum from the GS matches the experimental one very well, as the intensity distribution of the two doublets is spot-on and only a moderate shift of the excitation energies needed to be applied.

3.7 C–I bond length dependence of XAS cross-section

Here we investigate the calculated XAS in the region of the CoIn located at $R_{\mathrm{CI}} = 2.21$ Å. We distinguish between the absorption of the two diabatic states by assuming that absorption at photon energies below 49.2 eV corresponds to the $\tilde{E}$ diabatic state and absorption above 49.2 eV corresponds to the $\tilde{B}$ diabatic state. This allows us to integrate over the respective photon energies and investigate the integrated cross section as a function of the C-I bond length along the two adiabats that participate in the CoIn. These are shown in Figure S12.

From the first two columns, we see that the absorption along the adiabatic PECs switches from one electronic character to another within one geometric step (0.3 pm) of the RASPT2 calculation as we cross the CoIn. Such a sharp switch is necessitated by the fact that coupling between the $\tilde{B}$ ($^2\mathrm{A}_2$) and $\tilde{E}$ ($^2\mathrm{A}_1$) states is entirely symmetry-forbidden in the $\mathrm{C}_{3\mathrm{v}}$ point group (see Section 5).

The sum of the re-weighted cross-section (accounting for the different cross-section of the two diabatic states) is, however, left unchanged. The final column demonstrates the insensitivity of the absorption to the C-I bond length. The total absorption of the $\tilde{B}$ and $\tilde{E}$ states is almost constant from the FC point ($R_{\mathrm{CI}} = 2.14$ Å) all the way up to 2.24 Å at which point the absorption strength of the $\tilde{B}$ state starts to fall as the molecule starts to dissociate. These results therefore show that the transient absorption of the spectral regions attributed to the $\tilde{B}$ and $\tilde{E}$ states in the main text can be used as a reliable measure of their diabatic population.

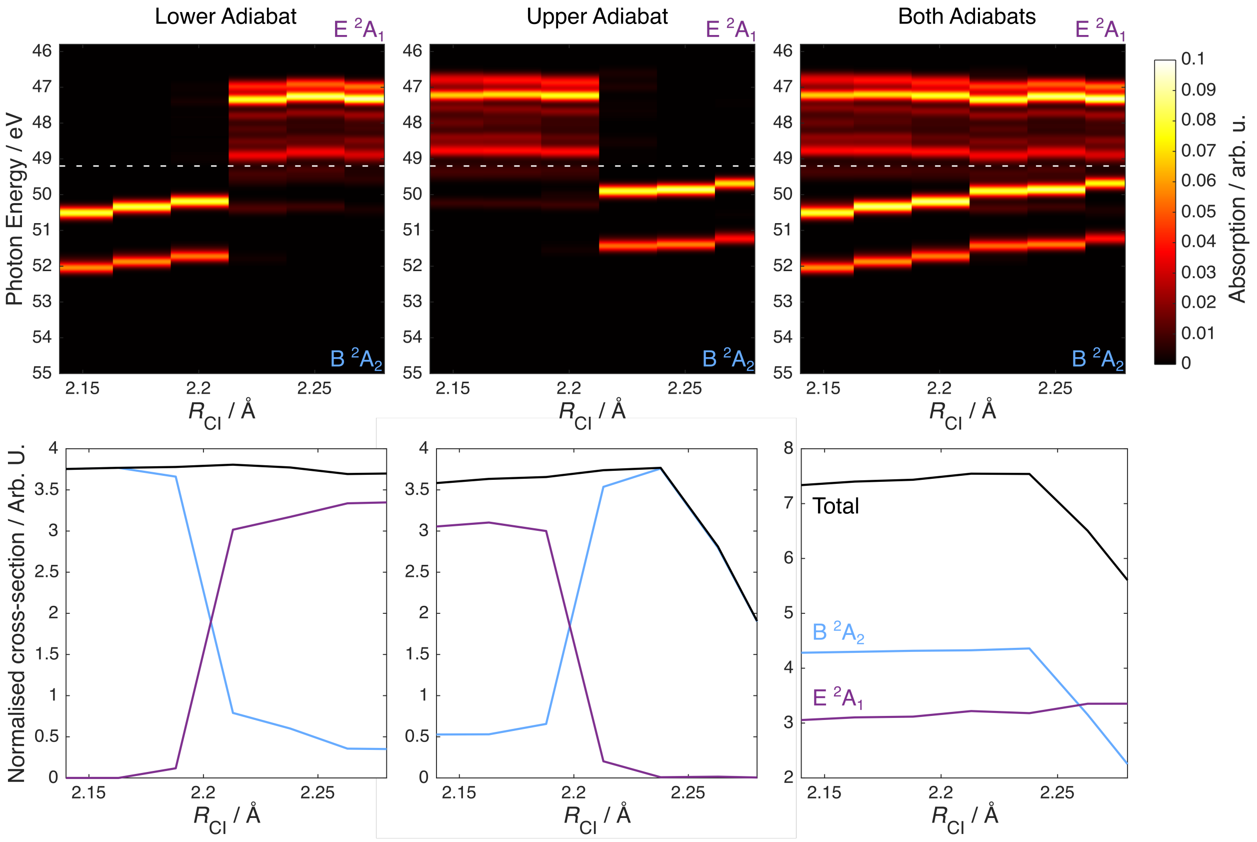

Figure S12. XAS as a function of C-I bond length in the region of the CoIn. The upper panels show the calculated cross sections as a function of the C-I bond length of the adiabatic states as well as the sum of the two. A dotted horizontal line indicates the energy that separates the absorption features of the $\tilde{B}$ and $\tilde{E}$ states. The lower panels show the result of integrating the upper and lower halves of the spectra shown in the upper panels. The higher energy half is multiplied by 1.85 to correct for the different integrated cross-sections of the $\tilde{B}$ and $\tilde{E}$ states.

4 Discussion on the absence of the Ã-state signal

Identifying or excluding the presence of the Ã-state in the experimental results is significantly easier if one has an estimate of the timescale of this state's dynamics. To obtain this we have performed quantum dynamical simulations of the state's vibrational dynamics by solving the time-dependent Schrödinger equation (TDSE)

$$i\hbar\frac{\partial}{\partial t}\chi(R,t) = \hat{H}\chi(R,t), \tag{S4}$$

$$\text{with } \hat{H} = -\frac{1}{2m_R}\frac{\partial^2}{\partial R^2}+\hat{V}_X(R), \tag{S5}$$

where m_r is the reduced mass along the dissociation coordinate R and $\hat{V}_X(R)$ is the potential energy operator. The numerical propagation on the adiabatic PEC is performed by integration of the TDSE according to

$$\chi(t+dt) = e^{(-i\hat{H}dt)}\chi(t) = \hat{U}\chi(t). \tag{S6}$$

The evolution operator $\hat{U}$ is expanded in a Chebyshev series[30]. The PEC used is represented on a one-dimensional spatial grid with 256 grid points and obtained by interpolating the results of the relaxed scan of cationic trifluoroiodomethane (S10). Due to the anti-bonding character of the Ã state, this is entirely repulsive. The limits of the grid used are 1.6 Å and 3.4 Å, respectively, and a Butterworth filter[31] is employed, which absorbs the parts of the wavepacket that reach the dissociation area. Loss of population in the simulations can then be directly associated with dissociation. The filter is of "left-pass" type (absorbing all parts on the right side of the grid) and placed at 3.3 Å with an order of 100. For the simulation a time step of 2 a.u. is used, and the simulation time is 250 fs. The propagation is initialized as the first eigenfunction of the neutral ground state potential to the $\tilde{A}$ state potential, i.e. assuming delta-pulse ionization. The quantum dynamical simulations are conducted with QDng a program developed in-house[32].

Figure S13 shows the change in population of the $\tilde{A}$ state over the 250 fs of simulation time. Within about 70 fs the $\tilde{A}$ state completely dissociates.

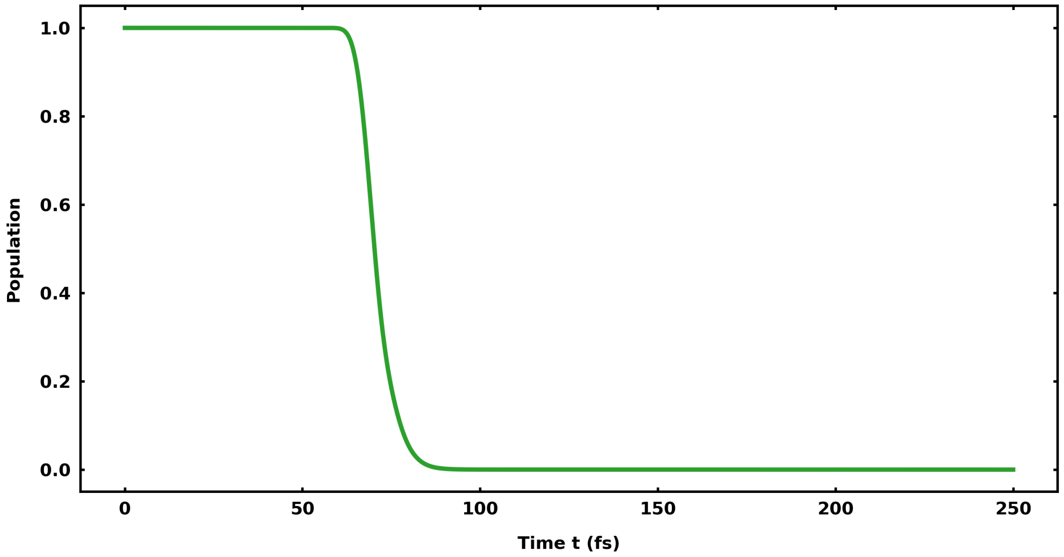

Figure S13. Population of the $\tilde{A}$ state of the CF_3I^+ cation over the 250 fs of simulation time.

Turning now to the experimental results, we find no transient signals which exhibit both such a timescale and matching the calculated XAS. Although the absorption feature at 46.1 eV shows spectral overlap with the largest peak of the theoretical Ã-state spectrum close to the FC point (see Figure 2F), its temporal evolution matches that of the $\tilde{X}_{1/2}$ state far better — it exhibits a very slight red shift on a 100 fs timescale. The Ã-state should instead exhibit a rapid blue-shift, on the previously-identified 70 fs timescale, converging to 46.70 eV — the absorption of the iodine $^2P_{1/2}$ state (see Fig. S14). For this reason it is not assigned to the Ã state.

We suggest two explanations for the absence of significant Ã-state absorption in our experimental results. The first reason combines the dependence of the strong-field ionization probability on the relative orientation of a molecule with the fact that the Ã-state's XAS is dominated by parallel transitions. Alignment through orientation-sensitive ionization is a phenomenon that has been identified in ATAS before, for example, in bromomethane[33]. If the Ã-state were to be created perpendicular to the pump and probe beam's polarization axis, the XUV probe would be almost blind to the presence of the population, explaining why the Ã state signal is not observed.

A second explanation stems from the fact that the strong-field ionization process is very difficult to

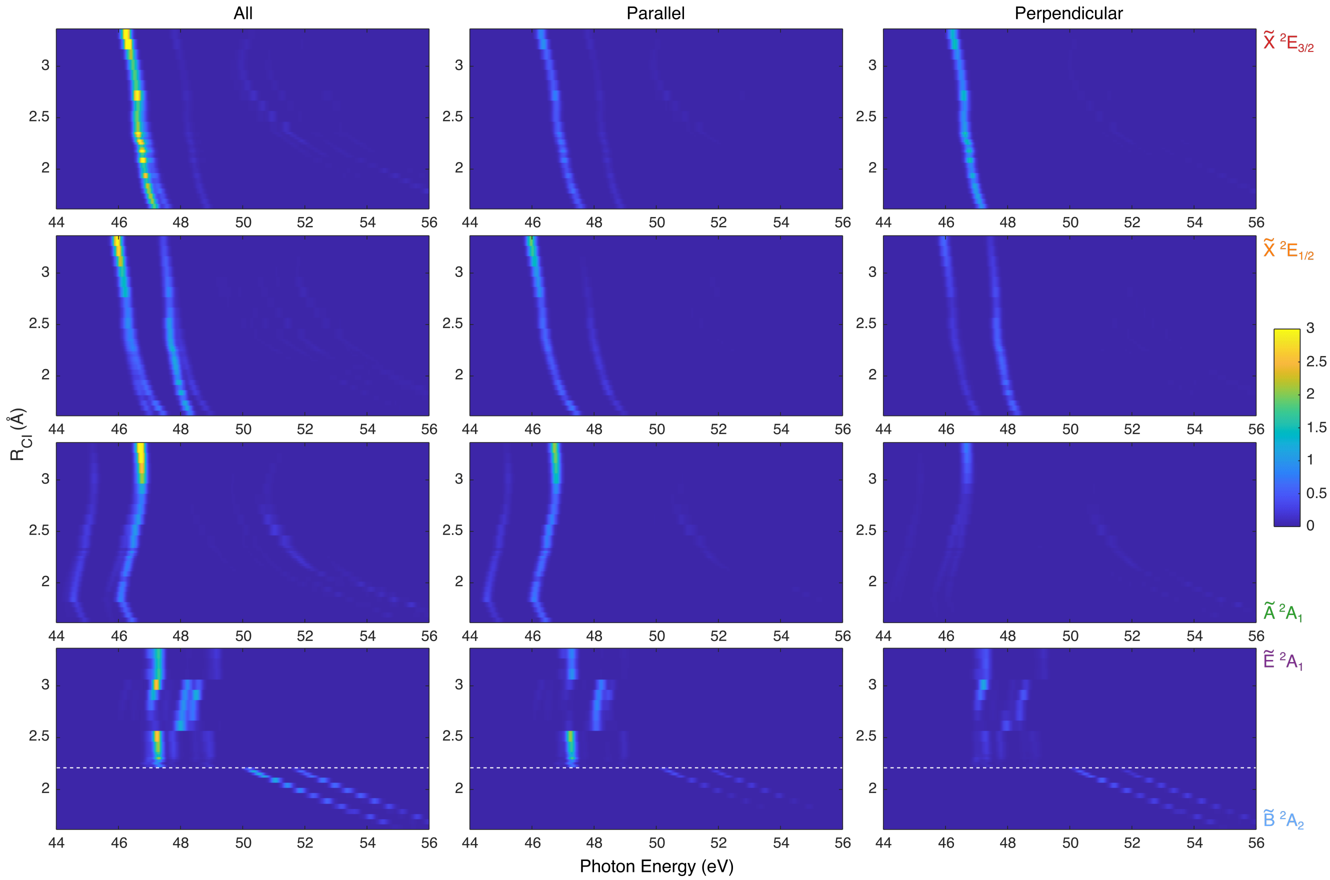

Figure S14. State- and orientation-resolved XAS of the first four adiabatic states. A common colorscale is used for all panels. The panels are labeled according to the molecule's orientation above, and according to their state on the right. The bottom panel is split at the CoIn ($R_{\mathrm{CI}} = 2.21$) according to its electronic character by a dashed white line

predict accurately, especially in the presence of spin-orbit coupling, multiple ionization channels, and when the parent population is significantly depleted (as is the case in our experiment). Under such conditions, the probability of creating the Ã-state in the first place may be significantly suppressed, leading to a lack of a measurable signal.

The correct explanation can be uncovered with further ATAS measurements which rotate the pump-polarization relative to that of the probe, or by introducing a third laser pulse to impulsively align the CF_3I prior to the ATAS experiment. Both of these efforts are currently underway but fall outside the scope of this work.

5 Symmetry analysis of the CT reaction

In this section we shall make use of symmetry arguments to draw conclusions about the number and the symmetry of the states and vibrational modes involved in the CT-reaction. This will allow us to infer the reaction path and explain the RASPT2 and DFT/MRCI PECs and those of the three-state model.

First, let us consider the vibrational problem. The point group of the neutral CF_3I molecule, as well as that of its cationic ground state is C_{3v}. The nine vibrational modes of the system consist of three doubly-degenerate E modes and three singly degenerate A_1 modes. CF_3I does not possess any A_2-symmetry

modes.

The RASPT2 and DFT/MRCI calculations reveal the symmetry of the cationic electronic states involved in the CT reaction. We find the donor $\tilde{B}$ state to be of $^2A_2$ symmetry and the acceptor $\tilde{E}$ state to be of $^2A_1$ symmetry. The RASPT2 and DFT/MRCI calculations also reveal the leading electronic character of the states (see Table S4 and Figure 2B of the main text), which show that the transition between $\tilde{B}$ and $\tilde{E}$ requires the rearrangement of two electrons. As we experimentally observe a non-zero population transfer delay, we conclude that the CT-reaction passes through one (or several) intermediate diabatic state(s), collectively labeled $\tilde{I}$. By splitting up the rearrangement into two single electron transitions we can identify the leading electronic character of the possible intermediate states, which we find to be of $(lp_3)^1(\sigma_4)^2(lp_{1,2})^3(\sigma_5^*)^1$ and $(lp_3)^2(\sigma_4)^2(lp_{1,2})^3(\sigma_5^*)^0$, as outlined in Section 3.5. Both of these configurations can only yield states of ^2E symmetry, informing us of the symmetry of the $\tilde{I}$ state(s).

Having identified the symmetry of all three diabatic states ($^2A_2$, ^2E, $^2A_1$), we can infer the symmetry of the vibrational modes that might couple them. As the three states are of different symmetries, their direct coupling is not possible by any totally symmetric (A_1) vibrational mode, such as the C-I stretching mode, at C_{3v} geometries. The reaction must therefore occur with participation of the remaining E vibrational modes.

eWhile the E vibrational modes are able to couple $\tilde{B}$ to $\tilde{I}$ and $\tilde{I}$ to $\tilde{E}$, linear vibronic coupling between $\tilde{B}$ and $\tilde{E}$ mediated by an E-symmetry mode is forbidden because[34,35]

$$A_1 \otimes A_2 = A_2 \neq E$$

and it is also forbidden by quadratic vibronic coupling by an E-symmetry mode because the symmetric part of $E \otimes E$ does not contain the direct product (A_2) of the irreducible representations of the $\tilde{B}$ and $\tilde{E}$ states:

$$[E \otimes E] = A_1 \oplus E \not\supset A_2.$$

The lowest order of vibronic coupling that allows for direct coupling of $\tilde{B}$ and $\tilde{E}$ is a bilinear coupling, requiring the simultaneous involvement of two E vibrational modes. The high-order nature of this interaction lends further support to the assumption that the direct coupling element V_{13} in our effective one-dimensional three-state model is negligibly small.

Compared to bilinear direct coupling, the indirect three-state coupling presented in our work is of lower order (requiring only a linear coupling in the E mode) and is the only mechanism that can reproduce a population transfer delay. From our symmetry analysis we can conclude that the one-dimensional reaction coordinate x in Figure 5 represents a reaction path that runs along the A_1 C–I stretching mode but includes a finite displacement along one or several E-symmetry vibrational mode(s) such that it misses the CoIn.

Since the RASPT2 caluclations are performed along a coordinate that preserves the C_{3v} geometry of the ionic ground state, the $\tilde{B}$/$\tilde{E}$ adiabats cannot contain the $\tilde{I}$ intermediate state character (and exhibit a very sudden change of character at the CoIn). The 3-model system adiabats, on the other hand, are not bound to the C_{3v} point group or required by symmetry to exhibit a discontinuity in their electronic character. Coupling to the intermediate state in the three-state model is allowed; as a result, they exhibit a more gradual change of electronic character and considerable NAC.

Until this point, the vibronic-coupling analysis has been formulated in the C_{3v} point group. However, this does not imply that it is only valid for C_{3v} geometries. CF_3I has a complete nuclear permutation inversion (CNPI) group given by the direct product of the S_3 permutation group and the inversion group: $S_3 \otimes E,E^*$. (For details, see[36]). The CNPI group contains a number of operations that possess a very large energy barrier (e.g. the inversion of the molecule). These energy barriers exceed the kinetic energy

available for molecular dynamics in our experiments and are such that they correspond to unfeasible operations. Keeping only the feasible operations leaves us with the operations of the $C_{3v}(M)$ group, the character table of which can be found on p. 672 of Ref.[36]. The molecular symmetry group $C_{3v}(M)$ is isomorphic with the C_{3v} point group. Therefore, the symmetry arguments formulated for the C_{3v} point group in this section also apply to the $C_{3v}(M)$ group, which describes CF_3I in all nuclear configurations (geometries) accessible under our experimental conditions, not only the $C_{3v}(M)$-symmetric ones. As a consequence, the vibronic coupling arguments remain unchanged when considering distorted geometries and working with the molecular-symmetry group $C_{3v}(M)$.

Supplementary references

1. Vutha, A. C. A simple approach to the Landau-Zener formula. *Eur. Journal Physics* **31**, 389–392, DOI: 10.1088/0143-0807/31/2/016 (2010). 1001.3322.
2. Hosking, J. & Wallis, J. Lognormal distribution. In *Regional frequency analysis: an approach based on L-moments*, chap. A.8, 197–199 (Cambridge University Press, 1997).
3. Matselyukh, D. T. *The Attosecond Electron Dynamics of Charge Migration and Charge Transfer: Opening the Door to Attochemistry*. Ph.D. thesis, ETH Zurich (2023).
4. Crane, S. W., Lee, J. W. & Ashfold, M. N. Multi-mass velocity map imaging study of the 805 nm strong field ionization of CF3I. *Physical Chemistry Chemical Physics* **24**, 18830–18840, DOI: 10.1039/d2cp02449g (2022).
5. Matselyukh, D. T., Despré, V., Golubev, N. V., Kuleff, A. I. & Wörner, H. J. Decoherence and revival in attosecond charge migration driven by non-adiabatic dynamics. *Nature physics* **18**, 1206–1213 (2022).
6. Faccialà, D., Toulson, B. W. & Gessner, O. Removal of correlated background in a high-order harmonic transient absorption spectra with principal component regression. *Optics Express* **29**, 35135–35148 (2021).
7. Frisch, M. J. *et al.* Gaussian 16 rev. a.03 (2016).
8. Chai, J.-D. & Head-Gordon, M. Long-range corrected hybrid density functionals with damped atom-atom dispersion corrections. *Phys. Chem. Chem. Phys.* **10**, 6615–6620, DOI: 10.1039/b810189b (2008).
9. Glukhovtsev, M. N., Pross, A., McGrath, M. P. & Radom, L. Extension of gaussian-2 (g2) theory to bromine and iodinecontaining molecules: Use of effective core potentials. *J. Chem. Phys.* **103**, 1878–1885, DOI: 10.1063/1.469712 (1995).
10. Krishnan, R., Binkley, J. S., Seeger, R. & Pople, J. A. Self-consistent molecular orbital methods. XX. a basis set for correlated wave functions. *J. Chem. Phys.* **72**, 650–654, DOI: 10.1063/1.438955 (1980).
11. Pritchard, B. P., Altarawy, D., Didier, B., Gibson, T. D. & Windus, T. L. New basis set exchange: An open, Up-to-Date resource for the molecular sciences community. *J. Chem. Inf. Model.* **59**, 4814–4820, DOI: 10.1021/acs.jcim.9b00725 (2019).
12. Schuchardt, K. L. *et al.* Basis set exchange: a community database for computational sciences. *J. Chem. Inf. Model.* **47**, 1045–1052, DOI: 10.1021/ci600510j (2007).
13. Feller, D. The role of databases in support of computational chemistry calculations. *J. Comput. Chem.* **17**, 1571–1586 (1996).

14. Fdez Galván, I. *et al.* OpenMolcas: From source code to insight. *J. Chem. Theory Comput.* **15**, 5925–5964, DOI: 10.1021/acs.jctc.9b00532 (2019).

15. Aquilante, F. *et al.* Modern quantum chemistry with [Open]Molcas. *J. Chem. Phys.* **152**, 214117, DOI: 10.1063/5.0004835 (2020).

16. Roos, B. O. *et al.* New relativistic atomic natural orbital basis sets for lanthanide atoms with applications to the ce diatom and LuF3. *J. Phys. Chem. A* **112**, 11431–11435 (2008).

17. Roos, B. O., Lindh, R., Malmqvist, P.-Å., Veryazov, V. & Widmark, P.-O. New relativistic ANO basis sets for actinide atoms. *Chem. Phys. Lett.* **409**, 295–299 (2005).

18. Roos, B. O., Lindh, R., Malmqvist, P.-A., Veryazov, V. & Widmark, P.-O. New relativistic ANO basis sets for transition metal atoms. *J. Phys. Chem. A* **109**, 6575–6579 (2005).

19. Roos, B. O., Lindh, R., Malmqvist, P.-Å., Veryazov, V. & Widmark, P.-O. Main group atoms and dimers studied with a new relativistic ANO basis set. *J. Phys. Chem. A* **108**, 2851–2858 (2004).

20. Roos, B. O., Veryazov, V. & Widmark, P.-O. Relativistic atomic natural orbital type basis sets for the alkaline and alkaline-earth atoms applied to the ground-state potentials for the corresponding dimers. *Theor. Chem. Acc.* **111**, 345–351 (2004).

21. Grimme, S. & Waletzke, M. A combination of KohnSham density functional theory and multi-reference configuration interaction methods. *J. Chem. Phys.* **111**, 5645–5655, DOI: 10.1063/1.479866 (1999).

22. Neville, S. P. & Schuurman, M. S. A perturbative approximation to DFT/MRCI: DFT/MRCI(2). *J. Chem. Phys.* **157**, 164103, DOI: 10.1063/5.0118285 (2022).

23. Costain, T. S., Ogden, V., Neville, S. P. & Schuurman, M. S. A DFT/MRCI Hamiltonian parameterized using only ab initio data: I. valence excited states. *J. Chem. Phys.* **160**, 224106, DOI: 10.1063/5.0210897 (2024).

24. Neville, S. & Schuurman, M. GRaCI: General Reference Configuration Interaction (2021).

25. Jin, Y. & Bartlett, R. J. Accurate computation of X-ray absorption spectra with ionization potential optimized global hybrid functional. *J. Chem. Phys.* **149**, 064111, DOI: 10.1063/1.5038434 (2018).

26. Kowalewski, M. & Seeber, P. Sustainable packaging of quantum chemistry software with the Nix package manager. *Int. J. Quantum Chem.* **122**, DOI: 10.1002/qua.26872 (2022).

27. Yates, B. W., Tan, K. H., Bancroft, G. M. & Tse, J. S. A variable energy photoelectron study of the valence levels and I 4d core levels of CF3I. *J. Chem. Phys.* **85**, 3840–3850, DOI: 10.1063/1.450904 (1986).

28. Rott, F. *et al.* Ultrafast strong-field dissociation of vinyl bromide: An attosecond transient absorption spectroscopy and non-adiabatic molecular dynamics study. *Struct Dyn* **8**, 034104, DOI: 10.1063/4.0000102 (2021).

29. O'Sullivan, G., McGuinness, C., Costello, J. T., Kennedy, E. T. & Weinmann, B. Trends in 4 <i>d</i> -subshell photoabsorption along the iodine isonuclear sequence: I, I + , and I 2 +. *Physical Review A* **53**, 3211–3226, DOI: 10.1103/PhysRevA.53.3211 (1996).

30. TalEzer, H. & Kosloff, R. An accurate and efficient scheme for propagating the time dependent schrödinger equation. *The Journal Chemical Physics* **81**, 3967–3971, DOI: 10.1063/1.448136 (1984).

31. Butterworth, S. On the theory of filter amplifiers. *Wireless Engineer* **7**, 536–541 (1930).

32. Kowalewski, M. & de Vivie-Riedle, R. QDng: A Grid Based Molecular Quantum Dynamics Package, DOI: 10.5281/zenodo.10944497 (2024).

33. Timmers, H. *et al.* Disentangling conical intersection and coherent molecular dynamics in methyl bromide with attosecond transient absorption spectroscopy. *Nature Communications* **10**, 1–8, DOI: 10.1038/s41467-019-10789-7 (2019).

34. Domcke, W., Yarkony, D. R. & Köppel, H. (eds.) *Conical intersections: Electronic structure, dynamics and spectroscopy*, vol. 15 of *Adv. Ser. in Phys. Chem.* (World Scientific, Singapore, 2004).

35. Wörner, H. J. & Merkt, F. Jahn-teller effects in molecular cations studied by photoelectron spectroscopy and group theory. *Angewandte Chemie International Edition* **48**, 6404–6424, DOI: 10.1002/anie.200900526 (2009).

36. Bunker, P. R. & Jensen, P. *Molecular symmetry and spectroscopy*, vol. 46853 (NRC research press, 2006).
